# A novel integrated injection snare for single-device endoscopic mucosal resection

**DOI:** 10.1055/a-2915-9753

**Published:** 2026-08-04

**Authors:** Xiaolong Rao, Guanyi Liu, Shibo Song, Bin Zhou, Yunlong Cai, Yuan Tian, Long Rong

**Affiliations:** 1 Endoscopy Center 26447 Peking University First HospitalBeijingChina

Endoscopic mucosal resection (EMR) is widely used to resect benign and early malignant gastrointestinal lesions worldwide. 1​ The traditional procedure of EMR includes submucosal injection with an injection needle followed by lesion resection with an electrosurgical snare. 2​ Sequential instrument exchange is complicated and time-consuming, especially for challenging lesions.

A novel integrated injection snare designed by Dr. Rao unifies submucosal injection and resection in a single device. The injection needle and electrosurgical snare traverse separate lumens within a dual-lumen sheath, connecting to a control handle at the proximal end. The control handle enables independent actuation of each component from the sheath's distal terminus. Single-handed ergonomic control provides full device maneuverability ( Fig.1 ; Video 1​ , Part 1).

**Fig. 1:**
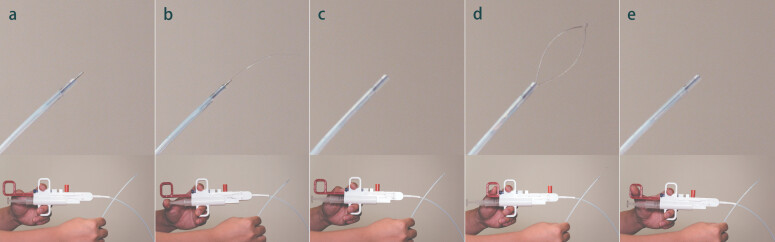
A single-handed control method for the integrated injection snare: ( **a** ) needle advancement, ( **b** ) liquid injection, ( **c** ) needle retraction, ( **d** ) snare deployment, and ( **e** ) resection.

**Video 1** Demonstration of a novel integrated injection snare, including instrument manipulation, ex vivo EMR in porcine colon model, in vivo conventional EMR, and in vivo pre-cut EMR.


Single-device EMR procedures in an ex vivo porcine colon model were first performed using this novel device ( Fig. 2 ; Video 1​ , Part 2). Furthermore, we supplemented clinical validation in real patients. Conventional EMR ( Video 1​ , Part 3) and pre-cut EMR ( Video 1​ , Part 4) were successfully performed on human colon lesions. An immediate transition from injection to snaring is achieved during EMR procedures, which reduces the risks of endoscope dislodgement and submucosal fluid diffusion. Interestingly, the one-handed operable control handle liberates the operator's other hand, enabling concurrent precise positioning of the device. All procedures were completed smoothly with stable intraoperative operation, confirming the clinical applicability and reliable performance of this integrated snare.

**Fig. 2:**
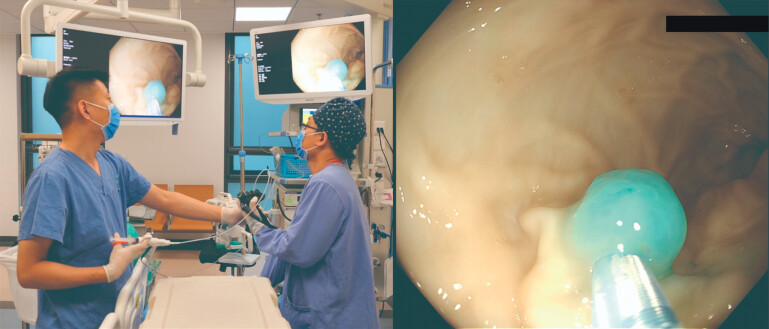
A still image extracted from Video 1​ , demonstrating electrosurgical resection with the novel integrated injection snare in an ex vivo porcine colon model.

All operators and assistants have reported that using this novel integrated device can reduce the time for instrument exchange, improve EMR procedural smoothness and operational convenience. The EMR wound surface of the novel device is similar to that of the traditional equipment. We confirmed the technical feasibility and operability of this novel integrated injection snare. Further studies to assess the superiority of this novel device will be conducted.

Endoscopy_UCTN_Code_TTT_1AQ_2AC

### Funding

National High Level Hospital Clinical Research Funding (Scientific and Technological Achievements Transformation Incubation Guidance Fund Project of Peking University First Hospital, Grant Nos. 2023CX03 and 2024CX16).

## References

[1] Hwang J H Konda V Abu Dayyeh B K Endoscopic mucosal resection Gastrointest Endosc 2015 82 02 215 226 26077453 10.1016/j.gie.2015.05.001

[2] Rashid M U Alomari M Afraz S EMR and ESD: Indications, techniques and results Surg Oncol 2022 43 101742 35370049 10.1016/j.suronc.2022.101742

